# Impact of School Gardens on Nutrition Education Among Limited‐Income Communities in Alabama

**DOI:** 10.1111/josh.13513

**Published:** 2024-10-26

**Authors:** Sofia O. Sanchez, Katie Funderburk, Erin Reznicek, Sondra M. Parmer, J. B. Hinnant

**Affiliations:** ^1^ Alabama Cooperative Extension System, Supplemental Nutrition Assistance Program—Education, 208 Duncan Hall Alabama Cooperative Extension System at Auburn University 36849 AL; ^2^ Alabama Cooperative Extension System, Supplemental Nutrition Assistance Program—Education, 205 Duncan Hall Auburn University 36849 AL; ^3^ Alabama Cooperative Extension System, Supplemental Nutrition Assistance Program—Education, 206 Duncan Hall Auburn University 36849 AL; ^4^ Alabama Cooperative Extension System, Supplemental Nutrition Assistance Program ‐ Education, 201 Duncan Hall Auburn University Auburn 36849 AL; ^5^ College of Human Sciences, Human Development & Family Science, 288 Spidle Hall Auburn University 36849 AL

**Keywords:** school garden, diet, nutrition policy, nutrition education, fruits and vegetables

## Abstract

**BACKGROUND:**

We sought to determine the impact of school gardens and nutrition education on student fruit and vegetable consumption from limited‐income communities in Alabama.

**METHODS:**

Third grade students at 99 Alabama schools with and without gardens on‐site were randomized to receive either nutrition education or to a wait‐list control group. Multi‐level models were used to assess the effects of treatment and control with and without school gardens.

**RESULTS:**

Students attending schools with gardens demonstrated highest consumption of fruits and vegetables prior to treatment. Nutrition education alone translated into significant dietary improvements. Both gardens and education translated into greatest dietary improvements.

**IMPLICATIONS FOR SCHOOL HEALTH POLICY, PRACTICE, AND EQUITY:**

Devising financially accessible, practical, and culturally relevant ways to improve student health through garden and nutrition education may help improve student diet. Forming comprehensive Extension, SNAP‐Ed, and school collaboration to support gardens, nutrition education, and healthy school policies and systems can be a way to support and sustain healthy student learning environments.

**CONCLUSIONS:**

Combined gardening and nutrition education is associated with increases in fruit and vegetable consumption among majority rural, Southern students from limited‐resource communities.

The US Dietary Guidelines recommend youth ages 2‐18 consume a variety of fruits and vegetables as part of a healthy dietary pattern to support optimal growth and development.^1^ Despite increases since 2003 in fruit and vegetable intake, as of 2023 almost one third (32.1%) of US youth do not eat a daily fruit, and almost half (49.1%) do not eat a daily vegetable.^2^, ^3^ These disparities are greater among limited‐resource, racial minority, and Alabama youth populations.^2^, ^4^ Therefore, strategies are needed to improve fruit and vegetable consumption among children.

Schools present ideal settings to engage youth in healthy eating behaviors due to factors such as peer modeling of fruit and vegetable consumption, the institutional setting providing structured routines for mealtimes, and the length of time spent in school.^5^, ^6^, ^7^ As such, prior work has used schools as a setting to provide students nutrition education and create supportive environments to encourage children to practice healthful habits.^8^, ^9^

Edible gardens increase availability and access to fresh produce for consumption.^10^ Therefore, introducing or implementing gardens as part of school‐based interventions has been studied as a method to improve fruit and vegetable consumption. Data demonstrate that youth with garden experiences exhibit increased knowledge of, preference for, and willingness to taste fruits and vegetables.^11^, ^12^, ^13^, ^14^, ^15^ Beyond dietary benefits, students experience beneficial impacts on general health, social and emotional well‐being, and academic performance.^16^, ^17^, ^18^, ^19^ These positive attitudes and behaviors in childhood can further extend into adolescence and adulthood.^16^, ^20^ Thus, the presence of school gardens in conjunction with nutrition education may augment positive behaviors among students compared to nutrition education alone, and some studies incorporate nutrition concepts into educational garden lessons^14^, ^21^, ^22^; though there are a lack of studies explicating these effects. Some studies demonstrate findings may vary by dose and frequency of the intervention, and community support.^23^, ^24^ Additionally, students experience inequitable access to school gardens, where gardens are less prevalent at schools serving students from non‐urban areas and from communities of lower socioeconomic position.^25^, ^26^ There is also a paucity of data on the effects of nutrition education on students with and without school garden participation, particularly in rural settings.

Body Quest (BQ) is a school‐based health promotion initiative designed to increase healthy eating among students from limited‐resource communities. Prior BQ interventions have resulted in increased self‐reported fruit and vegetable consumption among students and their parents.^27^, ^28^ However, the potential differential or additive effects of BQ in schools with and without gardens have not been determined. Therefore, the purpose of this study was to determine the impact of school gardens on BQ nutrition education among students from limited‐income communities in Alabama.

Based on prior data demonstrating gardening exposure supplemented with nutrition education increases fruit and vegetable consumption,^19^, ^29^ we hypothesized BQ would increase student self‐reported fruit and vegetable consumption when compared to the control group. We also hypothesized students attending schools with gardens would demonstrate increases in fruit and vegetable consumption when compared to students attending schools without gardens. Finally, we hypothesized students attending schools with both BQ education and school gardens would demonstrate highest increases in fruit and vegetable consumption when compared to students in the control group and without school garden access.

## METHODS

### Participants

During the 2018‐2019 school year, third graders attending public schools in Alabama comprised of at least 50% of students receiving free or reduced lunch through the National School Lunch Program were considered eligible and invited to participate in the intervention. The sample consisted of 4116 students attending 99 schools. In this sample, 51% of students were boys, 61% lived in a rural area, and 21% attended a school with a garden on‐site. See Table 1 for baseline participant characteristics. Participant race was self‐reported and obtained from school rosters. For these analyses, students were binned by white, black, and other; races other than white or black made up only 3.9% of the sample.

**Table 1:** Baseline Participant Characteristics

	n (%)
Gender	
Girl	2003 (48.7)
Boy	2113 (51.3)
Race	
White	2358 (57.3)
Black	1599 (38.8)
Other race	159 (3.9)
Ethnicity	
Hispanic	270 (6.6)
Non‐Hispanic	3846 (93.4)
County	
Urban	1599 (38.8)
Rural	2517 (61.2)
Condition	
Treatment	2219 (53.9)
Control	1896 (46.1)
Garden presence	
No garden	3137 (78.7)
Garden on‐site	847 (21.3)

Count and percentage of participant demographics at baseline.

Rural locations were determined based on the Alabama Rural Health Association county‐level definition of rurality, a composite score measuring population size per square mile as provided by the US Census, proportion of residents employed in a county, and dollar value of agricultural production per square mile.^30^

### Instrumentation

Dietary composition was assessed by asking students Likert scale‐like questions, with response options ranging from 0 = no times yesterday up to 4 = 4 or more times yesterday. Questions were based on the Supplemental Nutrition Assistance Program—Education (SNAP‐Ed) Evaluation Framework, and the complete instrument was previously described by Struempler et al.^27^, ^31^ To measure fruit and vegetable consumption, students were asked, “Yesterday, how many times did you eat vegetables?” and, “Yesterday, how many times did you eat fruit?” separately. Questions beyond fruit and vegetable consumption were excluded from this research question and analyses.

### Procedure

SNAP‐Ed Extension educators in this intervention served a 1‐ to 2‐county area. Within their counties, each educator recruited schools willing to participate and classrooms within schools, ensuring a total of at least 10 classrooms per educator participating in the intervention. Schools were randomly assigned to either a treatment or control group, with an educator's given classrooms split between treatment and control groups. After random assignment, educators administered the intervention.

The BQ curriculum was designed to meet Alabama Department of Education third grade standards, while promoting healthy foods, beverages, and physical activity in each lesson. Extension educators read recruitment scripts to third grade classes to explain participation in the program. Students took home forms for parents to sign and provide student assent, which students returned to school for the Extension educator to collect. Assented students took written pre‐ and post‐assessments evaluating nutrition and physical activity behaviors. Students in the treatment group received interactive education including iPad app education and vegetable tastings during the treatment period. Students in the control group received the same education, but only after the intervention period and post‐assessments were completed (ie, a waitlist control group).

On occasion, student lessons were held in the garden or incorporated garden produce. However, due to the varying capacity of schools to offer garden exposure, and because garden access was permitted outside of the BQ intervention, educators simply noted whether the garden was accessible to students or not.

### Data Analysis

Analyses were performed using SPSS 28 and MPlus 8.8 by a statistician. Independent variables were: BQ education (treatment or control group), garden (presence of a garden at a given school); gender (boy or girl), ethnicity (Hispanic or Non‐Hispanic), urbanicity (school located in a rural or urban county), and race (black/African American, white, or other). Dependent variables were fruit and vegetable consumption. All participant demographics were self‐reported. Students were grouped in the analyses as following: students in the treatment group and attending a school with a garden (TG), students in the control group and attending a school with a garden (CG), students in the treatment group and attending a school without a garden (TNG), and participants in the control group and attending a school without a garden (CNG).

Preliminary 1‐way analyses of variance (ANOVAs) were used to determine if there were baseline differences in fruit and vegetable consumption between students. Multi‐level models were used to test the study's hypotheses: whether BQ education, the presence of a school garden, or the interaction (ie, moderation) between BQ education and presence of a school garden predicted change in fruit and vegetable consumption pre‐ to post‐intervention. Fruit and vegetable consumption were considered as separate dependent variables but estimated in parallel (ie, simultaneously) in analyses. First, an unconditional model with no predictors was estimated to separate the total variance of fruit and vegetable consumption into within‐students (ie, repeated measures, pre‐ to post‐intervention), between‐students, and between‐schools. Intraclass correlation coefficients (ICCs), the proportion of variance at a given level of analysis, were evaluated with a value of >0.05 to justify multi‐level modeling.^32^ If the ICC <0.05 in fruit or vegetable consumption at a given level of analysis, that level was omitted from subsequent analyses. Next, the within‐student effect of time (ie, pre‐post fruit and vegetable consumption quantities) was estimated and considered fixed (ie, constant across higher levels of nesting) or random (ie, allowed to vary across higher levels of nesting). Finally, BQ education, the presence of a school garden, and the interaction between BQ education and presence of a school garden were added as predictors of fruit and vegetable consumption. Gender, ethnicity, race, and urbanicity were included in the models as covariates.

## RESULTS

### Baseline Differences

Results from 1‐way ANOVAs are presented as mean ± SD in Table 2. For fruit consumption, TG was significantly different (*p* < .001) from TNG and CNG, but not CG. TNG, CNG, and CG were not significantly different from each other. TG students reported the highest baseline fruit consumption (2.00 ± 1.47 times per day), followed by CG (1.84 ± 1.38 times per day), TNG (1.70 ± 1.42 times per day), and CNG (1.70 ± 1.43 times per day). This indicates that students attending schools with pre‐existing gardens demonstrated the highest fruit consumption at baseline, prior to any nutrition education. For vegetable consumption, TG was significantly different (*p* < .05) from TNG, but not from CG or CNG. TG students reported the highest baseline vegetable consumption (1.44 ± 1.41 times per day), followed by CG (1.46 ± 1.39 times per day), CNG (1.26 ± 1.33 times per day) and TNG (1.25 ± 1.31 times per day). Similar to fruit consumption, students with pre‐existing gardens demonstrated highest vegetable consumption at baseline, prior to any nutrition education.

**Table 2:** Changes in Fruit and Vegetable Consumption

	Pre‐BQ	Post‐BQ
	Mean (SD)	Mean (SD)
Fruit		
TG	2.00 (1.47)	2.11 (1.45)
CG	1.84 (1.38)	1.88 (1.31)
TNG	1.70 (1.42)	1.97 (1.36)
CNG	1.70 (1.42)	1.78 (1.41)
Vegetable		
TG	1.44 (1.41)	1.60 (1.32)
CG	1.46 (1.40)	1.57 (1.35)
TNG	1.25 (1.31)	1.61 (1.38)
CNG	1.26 (1.33)	1.30 (1.26)

BQ, body quest; TG, treatment group and school garden; CG, control group and school garden; TNG, treatment group and no school garden; CNG, control group and no school garden.

### Multilevel Model Results: 3‐Level Model

The initial 3‐level model demonstrated an ICC = 0.027 for vegetable consumption and an ICC = 0.029 for fruit consumption at the school level. Given the small amount of between‐school variance in fruit and vegetable consumption, the school level of analysis was omitted from subsequent models.

### Two‐Level Model

The 2‐level model demonstrated an ICC = 0.354 for fruit consumption and an ICC = 0.341 for vegetable consumption, indicating meaningful variability in fruit and vegetable consumption at both within‐student and between‐student levels of analyses that could be accounted for by BQ education, the presence of a school garden, or the interaction between BQ education and presence of a school garden. Therefore, results were interpreted from the 2‐level model. Results are presented in Table 3.

**Table 3:** Multilevel Model Results of Daily Fruit and Vegetable Consumption (N = 4116 Individuals From 99 Schools)

	Daily Fruit and Vegetable Consumption (Times Per Day)							
	Fruit Intercept		Fruit Slope		Vegetable Intercept		Vegetable Slope	
Predictors	Unstandardized	SE	Unstandardized	SE	Unstandardized	SE	Unstandardized	SE
	Coefficient		Coefficient		Coefficient		Coefficient	
Between students (level 2)	1.599**	0.050	0.063	0.060	1.222**	0.047	0.055	0.056
BQ education	−0.039	0.051	0.201*	0.064	−0.038	0.048	0.347**	0.059
Garden	−0.036	0.097	−0.076	0.119	0.090	0.096	0.103	0.118
BQ education × garden interaction	0.355*	0.121	−0.106	0.146	0.092	0.118	−0.320*	0.141
Boy	−0.155*	0.045	0.005	0.055	−0.101*	0.043	−0.038	0.052
Hispanic	0.237*	0.102	−0.130	0.127	−0.007	0.095	0.098	0.121
Urbanicity	0.072	0.049	0.075	0.061	0.104*	0.046	−0.056	0.056
Black/African American	0.175	0.129	−0.373*	0.154	−0.017	0.126	−0.264	0.149
Other race	0.230	0.130	0.317*	0.154	0.183	0.126	0.256	0.149

Condition, treatment or control group; garden, presence of a garden at a given school; interaction, moderation effect of school gardens on the Body Quest education; gender, boy or girl, where boy is the referent group; ethnicity, Hispanic or Non‐Hispanic, where Non‐Hispanic is the referent group; African American/black, student self‐reported race, where white is the referent group; other race, students who self‐reported race as other than white or African American/black.

* *p* < .05.

** *p* < .01.

*** *p* < .001.

### Fruit Consumption

There was a significant interaction of condition and garden on pre‐intervention levels of fruit consumption between students (*β* = 0.335, *p* < .05) but no main effects for condition or garden (*p*s >.445). We found that TG consumed fruit 0.335 times more frequently than CNG students.

**Figure 1:**
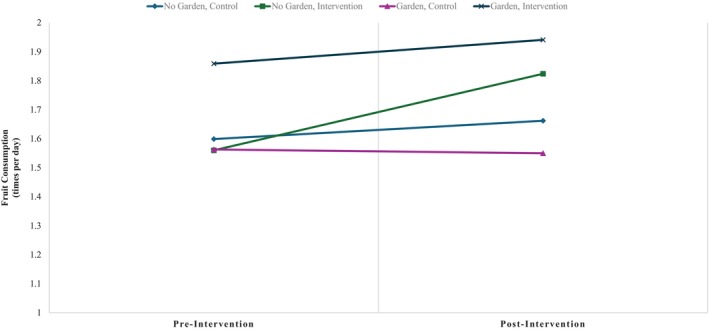
Differences in Fruit Consumption From Pre‐ to Post‐Intervention. Students in the garden and intervention group exhibited highest fruit consumption at baseline. Students in the no garden and intervention group exhibited highest increases in fruit consumption from pre‐ to post‐intervention.

Gender and ethnicity were significant predictors of fruit consumption between students at baseline (*β* = −0.155, *p* = .001; *β* = 0.237, *p* < .05), but rurality and race were not (*p*s >.07). This indicates that boys consumed fruit 0.155 times less frequently compared to girls, and that Hispanic students consumed fruit 0.237 times more frequently compared to non‐Hispanic. See Figure 1 for fruit consumption.

BQ education alone was a significant predictor of increases in fruit consumption pre‐ to post‐intervention (*β* = 0.201, *p* < .05); students receiving BQ education had greater increases in fruit consumption than those in the control group. Presence of a school garden and the interaction between BQ education and school garden were not predictive of changes in fruit consumption (*p*s >.465). Race was a significant predictor of change in fruit consumption over time. When compared to white students, black students or students identifying as another race demonstrated significant differences (*β* = −0.373, *p* < .05; *β* = 0.317, *p* < .05, respectively). Gender, ethnicity, rurality were not significant predictors, *p*s >.214. Pre‐intervention fruit consumption significantly covaried with changes in fruit consumption (*β* = −1.169, *p* < .001) in those students who consumed more fruit pre‐intervention demonstrated less change in their fruit consumption pre‐ to post‐intervention.

### Vegetable Consumption

BQ education, the presence of a school garden, or the interaction between BQ education and presence of a school garden did not predict pre‐intervention levels of vegetable consumption, *p*s >.428. Gender and rurality were significant predictors (*β* = −0.101, *p* < .05; *β* = 0.104, *p* < .05) but not ethnicity or race, *p*s >.148. Similar to results from fruit, boys demonstrated lower vegetable consumption, by 0.101 times per day, on average. Different from fruit consumption, urban students consumed vegetables 0.072 times more frequently compared to rural students.

**Figure 2:**
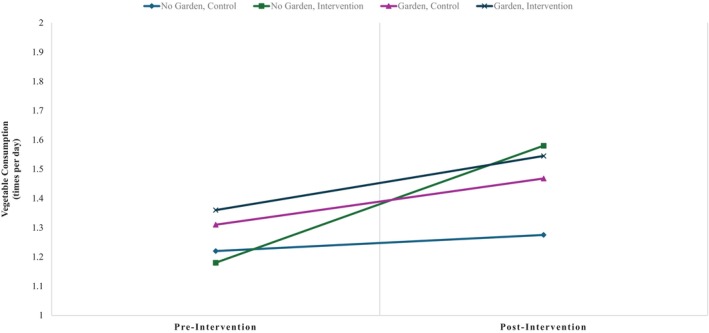
Differences in Vegetable Consumption From Pre‐ to Post‐Intervention. Students in the garden and intervention group exhibited highest fruit consumption at baseline. Students in the no garden and intervention group exhibited highest increases in fruit consumption from pre‐ to post‐intervention.

BQ education alone was a significant predictor of change in vegetable consumption pre‐ to post‐intervention (*β* = 0.347, *p* < .001) but presence of a school garden was unrelated to changes in vegetable consumption. Different from fruit, there was a significant interaction between BQ education and presence of a garden on change in vegetable consumption (*β* = −0.320, *p* < .05; see Figure 2). Gender, ethnicity, rurality, and race were not significant predictors, *p*s >.07. Vegetable consumption significantly covaried with its slope (*β* = −1.064, *p* < .001) in those students who consumed more vegetables pre‐intervention had less change in their vegetable consumption pre‐ to post‐intervention.

## DISCUSSION

These findings add to the literature describing the effects of nutrition education and gardening experiences on student dietary outcomes. Our findings demonstrate the presence of school gardens is associated with increased student fruit and vegetable consumption, prior to any nutrition‐related interventions. These higher baseline dietary patterns could be attributed to school garden exposure supporting positive health behaviors.

We also demonstrate that BQ nutrition education increased both fruit and vegetable consumption, supporting our primary hypothesis. Additionally, we found an interaction between BQ education and presence of a garden for changes in vegetable consumption. Students receiving BQ but who did not have access to a garden exhibited the greatest increases in vegetable consumption.

These findings align with national data demonstrating children consume fruit more frequently than vegetables.^2^ The data also support the idea of a “ceiling effect,” where children with increased liking of a certain food at baseline experience fewer increases after exposure to that food, compared to children with lower liking at baseline.^33^ Additionally, these findings align with national patterns of racial differences in youth fruit and vegetable consumption over time.^34^ In our data, relative to white students and students of other races, black students consumed fruit at a smaller rate of change pre‐to‐post. These findings may have greater implications for studying student vegetable consumption. A recent review of school gardens found that students in studies shorter in duration (ie, 12‐28 weeks) and with a smaller sample size demonstrate improvements specifically in vegetable intake.^23^ In our study, the intervention lasted 15 weeks, and the findings align with behavior change trends from the review previously cited.

Of the limited studies investigating gardening and nutrition education, findings are mixed. In a Southern, urban community, gardening in conjunction with nutrition education over 28 weeks resulted in significantly greater increases in vegetable consumption compared to nutrition education alone in second grade students.^29^ Similarly, among Midwestern, urban sixth graders, a school‐based gardening and nutrition intervention over 12 weeks resulted in significant increases in fruit and vegetable consumption when compared to nutrition education alone and when compared to the control group.^35^ This work expands upon these previous studies in demonstrating similar findings among third grade students in the Southeastern US, while adding to the limited body of literature surrounding nutrition education with gardening. The findings are particularly important as most participants in this sample resided in rural counties.

In contrast to our findings, one intervention found no significant differences in fruit or vegetable intake.^36^ However this was a 10‐week intervention in Australia, therefore length of intervention or difference in sociocultural factors may influence student intake. Indeed, studies suggest that other environmental factors are strong influences on student diet, such as home gardening or fruit and vegetable availability in the home.^37^, ^38^

Disparities in food access and body weight are greater for rural youth. Of students living in rural areas, 54% frequently or always purchased food from gas stations compared to 29% of students living in urban areas,^39^ a retail setting typically offering high calorie, high fat foods that are low in beneficial nutrients. Comparisons between urban and rural childhood obesity prevalence find children living in rural environments display higher prevalence and increased odds of overweight and obesity.^40^ Given these differences, BQ education has utility in teaching students in rural communities about healthful food choices as a method to improve diet. School gardens may be of greater value for rural children as this population has greater accessibility to agriculture due to proximity and cultural norms.^41^ Despite no difference in gardening interest based on rurality, students in rural areas report a greater degree of accessing food from gardens.^39^

Body Quest is an Alabama‐designed nutrition education program, and has been taught in underserved public schools for over 10 years. These findings provide rationale for adding garden‐based activities to the existing curriculum, particularly for students attending schools without adequate support for independently funded school gardens. While fruit and vegetable tastings are part of lesson content, integrating locally grown produce or produce grown directly from school gardens (ie, Farm to School) may provide greater benefit for students.

Despite improvements in US food insecurity, Alabama remains among the states with the highest food insecurity rates.^42^ School gardens may be explored as a tool to alleviate food insecurity among families with Alabama school‐aged children, although these effects may differ by household composition and demographics.^43^, ^44^ Beyond diet, a prior study of urban Alabama students found farm to school activities allowed opportunities for students to experience community engagement and build positive relationships,^45^ particularly among underserved students. These studies warrant further study of Alabama school gardens.

## IMPLICATIONS FOR SCHOOL HEALTH POLICY, PRACTICE, AND EQUITY

Activities related to school gardening fall under the umbrella term “farm to school,” defined as programming that offers at least one of the following: procuring and implementing opportunities to eat local produce, school gardening, or education related to health, nutrition, or agriculture.^46^ Establishing optimal farm to school practices, such as healthy school wellness policies encouraging gardening, enable environments conducive to healthy student growth and development.^47^, ^48^, ^49^ Healthy school environments are associated with healthy weight status, lower consumption of fast foods, increased water access and intake, and increased physical activity among students.^50^, ^51^, ^52^, ^53^ This is relevant as schools with farm to school programs are 4 times more likely to incorporate garden produce in cafeteria services, however, only 26%‐50% of Alabama schools operate an edible garden.^54^ This becomes especially important as the odds of having a farm to school program decreases as the percentage of students eligible for free and reduced price meals increases.^55^ Thus, devising financially accessible, practical, and culturally relevant ways to improve student health through garden and nutrition education may help improve student diet. Cooperative Extension offices are housed in or located near every county in the United States,^56^ offering a diverse portfolio of community services, such as master gardeners and SNAP‐Ed. Nationally, 65% of adults are aware of their state's Extension programming, and 23% have heard of but not used Extension services.^57^ Forming comprehensive Extension, SNAP‐Ed, and school collaboration to support gardens, nutrition education, and broadly healthy school policies and systems can be a way to support and sustain healthy student learning environments. Specific to this collaboration, BQ education was successful at increasing student self‐reported diet, particularly for students attending schools without school gardens. To see the greatest benefits to fruit and vegetable consumption, students would receive both BQ and exposure to gardens at their school.

There may be challenges in implementing BQ and school garden‐related policy. For example, a recent study found that 26% of schools operating a farm to school program do not continue after 1 year.^58^ Factors such as community assets (ie, health care or social services), presence of local agriculture, and school investment of an on‐site kitchen may be stronger predictors of farm to school participation.^59^ Schools located in communities with these characteristics may be ideal sites at which to implement such policy. Additionally, schools with diverse programming, such as a farm field trip and tastings in a lunchroom, are more likely to maintain farm to school participation into the future.^58^ Incorporating these activities into existing institutional systems and elementary curricula can help promote and sustain both BQ and school garden participation.

### Limitations

This study is not without limitations. First, garden size, type of produce grown, or exposure frequency were not captured in this study. Additionally, schools willing to host gardens may be more likely to implement other healthy practices. Future studies may consider measuring whether degree of garden success influences dietary outcomes.^26^ This study also utilizes self‐reported times fruits and vegetables were consumed in a day. However, the purpose of this study was to measure dietary patterns and not caloric intake. Therefore, we believe the findings are still valid given the implications for underserved communities.

### Conclusions

This work demonstrates that combined gardening and nutrition education is associated with positive increases in fruit and vegetable consumption among majority rural, Southern students from limited‐resource communities. The analysis between urban and rural students found no significant differences, suggesting BQ is useful in both types of environments. However, given the sample size was majority rural and due to persistent health disparities between urban and rural populations, much work still needs to be done in this area.

### Human Subjects Approval Statement

This study was approved by the Auburn University Institutional Review Board, protocol # 17‐289 MR 1707.

### Conflict of Interest

There are no conflicts of interest to report.

### Author Contributions

S.O.S: writing—original draft (lead); writing—review and editing (equal); data curation (equal). K.F.: methodology (supporting); review and editing (equal). E.R.: writing—review and editing (equal). S.P.: conceptualization; methodology; writing—review and editing (equal). J.B.H.: formal analysis (lead); software (lead); data curation (equal); writing—review and editing (equal).
